# Genetic tracing of HCoV-19 for the re-emerging outbreak of COVID-19 in Beijing, China

**DOI:** 10.1007/s13238-020-00772-0

**Published:** 2020-08-17

**Authors:** Jing Yang, Peihua Niu, Lijuan Chen, Liang Wang, Li Zhao, Baoying Huang, Juncai Ma, Songnian Hu, Linhuan Wu, Guizhen Wu, Chun Huang, Yuhai Bi, Wenjie Tan

**Affiliations:** 1 CAS Key Laboratory of Pathogenic Microbiology and Immunology, Institute of Microbiology, Center for Influenza Research and Early-warning (CASCIRE), CAS-TWAS Center of Excellence for Emerging Infectious Diseases (CEEID), Chinese Academy of Sciences, Beijing, 100101 China; 2 National Institute for Viral Disease Control and Prevention, Chinese Center for Disease Control and Prevention (China CDC), Beijing, 102206 China; 3 Beijing Center for Diseases Prevention and Control, Beijing, 100013 China; 4 Center for Biosafety Mega-Science, Chinese Academy of Sciences, Wuhan, 430071 China; 5 University of Chinese Academy of Sciences, Beijing, 101408 China

The ongoing pandemic of coronavirus disease 2019 (COVID-19) caused by a novel severe acute respiratory syndrome coronavirus 2 (SARS-CoV-2, also named as 2019-nCoV or HCoV-19) poses an unprecedented threat to public health (Zhu et al., 2020; Wang et al., 2020; Jiang et al., 2020). The novel HCoV-19 virus has rapidly spread into multiple countries across the world since it was first reported in December 2019. The World Health Organization (WHO) declared COVID-19 as a pandemic on 11th March 2020. As of 4th July, over 10 million confirmed COVID-19 cases have been reported in over 200 countries/regions with more than 0.5 million deaths, including 85,287 documented cases and 4,648 deaths in China (WHO, 2020a).

Since the emergence of HCoV-19, Chinese government employed a series of rapid and effective non-pharmaceutical interventions, such as travel restriction, social distancing, extending holidays, and postponing large public events and mass gathering, to contain COVID-19 and prevent more infections (Lai et al., 2020). By the end of February 2020, the domestic spread of COVID-19 in China has been basically controlled but the epidemic situation outside China is still severe (WHO, 2020a). Since imported cases were sporadically reported, domestic resurgence caused by imported infections is likely in China (http://www.nhc.gov.cn/xcs/yqtb/list_gzbd.shtml; Li et al., 2020). Very recently, after no new case reported for 56 consecutive days, a confirmed COVID-19 case was identified in Beijing on 11th June, 2020 (http://www.nhc.gov.cn/xcs/yqtb/list_gzbd.shtml). Subsequently, a total of 334 COVID-19 cases were gradually confirmed by 4th, July 2020, and most of them have an exposure history in Xinfadi wholesale market, Beijing (WHO, 2020b). To reveal the potential genetic source and transmission paths of HCoV-19 causing this outbreak can help strengthen control measures to prevent such incidents in Beijing or other regions in the world.

In this study, we collected clinical specimens from both patients and environments in Xinfadi wholesale market, and the specimens were individually processed and sequenced using nanopore and MiSeq system. 16 consensus sequences of HCoV-19 were generated for short sequencing reads mapped to a reference strain EPI_ISL_402119 from GISAID (https://www.gisaid.org/) in CLC Genomics Workbench 20.0.3.0 and later were submitted to China National Microbiology Data Center (NMDC; http://nmdc.cn/coronavirus; Accession id: NMDC60013485-NMDC60013500). All HCoV-19 sequences isolated from Xinfadi wholesale market were recognized as lineage B1.1 using PANGOLIN (https://github.com/hCoV-2019/pangolin). The lineage B1.1 was mostly circulating in Europe, and 8,822 sequences of lineage B1.1 were submitted to GISAID as of June 18, 2020, including sequences from England (6,924/78.49%), the United States of America (240/2.72%), Australia (200/2.27%), Belgium (147/1.67%), Netherlands (137/1.56%), and Iceland (128/1.45%) (https://github.com/cov-lineages/lineages). Few viruses of lineage B1.1 were isolated from mainland China and most of them were collected from imported cases before this outbreak. Further, HCoV-19 isolated in mainland China was genetically distant to these from Xinfadi wholesale market (Fig. 1A). The phylogenetic analyses and lineage assignment suggested that the virus isolated from Xinfadi wholesale market in the outbreak in Beijing may be imported from abroad.

**Figure 1:**
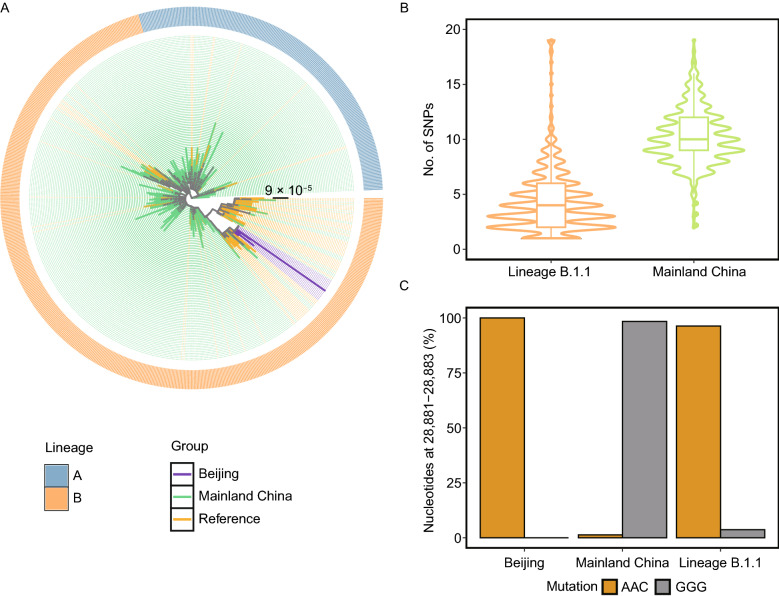
Phylogenetic and molecular characteristics of HCoV-19 viruses isolated from Beijing in June 2020, mainland China, and lineage B1.1. (A) Phylogeny of HCoV-19 among isolates from the re-outbreak of Beijing in June, mainland China (except the re-outbreak of Beijing in June), and representative viruses in different lineages. (B) The number of SNPs between isolates from Beijing and lineage B1.1, and between isolates from Beijing and other genomes from mainland China, respectively. (C) The proportion of GGG and AAC mutations at 28,881–28,883 sites of viruses isolated in Beijing, mainland China, and lineage B1.1

Furthermore, we counted the frequency of SNPs among HCoV-19 from Xinfadi wholesale market, mainland China (except isolates in this outbreak), and lineage B1.1 (Fig. 1B). More SNPs were found in virus genomes between Xinfadi wholesale market and mainland China, compared to those between Xinfadi wholesale market and lineage B1.1. Further, three consecutive base mutations (GGG mutated to AAC) at position 28,881–28,883 (according to coordinate of reference strain, MN908947.3) were detected in all HCoV-19 isolates from Xinfadi wholesale market (Fig. 1C). This mutation pattern was first detected in England on 15th February, 2020, and was a key molecular characteristic of lineage B1.1. The molecular characteristics of HCoV-19 further indicated that the virus causing Beijing’s outbreak might be introduced from abroad rather than from local. Five replicates of Bayesian phylodynamic inferences of HCoV-19 from Xinfadi wholesale market and lineage B1.1 (randomly selecting 10 sequences per week from lineage B1.1) were performed to infer the time of most recent common ancestor (TMRCA) of the genomes isolated in Beijing in June 2020 by BEAST 1.10.4 (Suchard et al., 2018). Our results are robust to different sampling datasets and indicated the virus causing the outbreak in Beijing may be firstly appeared on 6th May, 2020 (95% HPD: 2020-04-19, 2020-05-21).

Currently, the spread routes of HCoV-19 associated with Xinfadi wholesale market imported into Beijing remain uncovered. In addition to HCoV-19 isolated in environmental samples (chopping board and floor drain) from Xinfadi wholesale market in our study, several surfaces in the market were also tested positive for HCoV-19, including a cutting board in a booth handling imported salmon (Normile, 2020). Further, HCoV-19 could be highly stable in low temperature (decreasing only about 0.7 log-unit of infectious titer after 14 days at 4 °C) (Chin et al., 2020). One hypothesis is proposed that the virus may be imported by contaminated fish from Europe and transported via cold chain transportation. Another possible transmission mode may be that international travelers with asymptomatic symptoms or false negative of nucleic acid test spread the virus into China, and the virus was transmitted to the market by infected humans. The humid and chilled air and suboptimal hygienic conditions in the market may provide an ideal environment for amplification of virus, and subsequently the virus was spread by people working and visiting in the markets, resulting in the re-emerging outbreak of COVID-19 in Beijing (Normile, 2020).

In summary, strict strategies of monitor and quarantine for both international travelers and imported goods are essential to prevent the potential secondary extensive outbreak in China in the critical period of HCoV-19 still circulating worldwide. Implementing differentiated and location-specific prevention and control measures which tailored to local epidemic conditions could be an experience and lesson learned from China’s first phase of effective responses to COVID-19. Further, WHO played a crucial leading role to coordinate the global responses and build international expert networks to control and contain COVID-19 pandemic. International cooperation and collaboration to control the pandemic are needed to effectively fight and tackle the COVID-19 pandemic, with special attention to the vulnerable countries/regions by providing essential medicines, vaccines and medical equipment to combat COVID-19.

## Electronic supplementary material

Below is the link to the electronic supplementary material.

Supplementary material 1 (PDF 258 kb)
