# Supplementary material for: Genetic tracing of HCoV-19 for the re-emerging outbreak of COVID-19 in Beijing, China
Source: Protein Cell. 2020 Aug 17;12(1):4–6. doi: 10.1007/s13238-020-00772-0 (PMC7430935; doi:10.1007/s13238-020-00772-0)
Supplement: 13238_2020_772_MOESM1_ESM — Materials and Methods [file 13238_2020_772_moesm1_esm.pdf]

Materials and Methods

Sample collection and viral genome sequencing

The clinical specimens were collected from Xinfadi wholesale market in Beijing, including human cases and environmental samples. Specimen processing was in Biosafety level three (BSL-3) laboratory. Nucleic acids were extracted from 140 μl processed specimen using a QIAamp Viral RNA Mini Kit (QIAGEN, Cat no. 52904) according to manufacturer's instructions. Approximately 60 μl of total nucleic acid eluates for each sample was recovered into nuclease-free tubes, and either tested immediately or stored at -70 ℃.

All specimens included in the study were separately processed and sequenced using nanopore and MiSeq system. The nucleic acid in the sample was insufficient for library preparation and whole genome specific amplification was used to increase the amount of nucleic acid extracted from the sample. Amplification products were purified with the QIAquick PCR Purification Kit (QIAGEN, Cat no. 28104), and used for Illumina library preparation with the Nextera XT library prep kit. Libraries were barcoded with non-overlapping dual indexes, pooled and sequenced using the Illumina NextSeq 550 platform. We got a total of 16 consensus sequences of HCoV-19 for short sequencing reads mapped to a reference strain EPI_ISL_402119 from GISAID database (https://www.gisaid.org/) in CLC Genomics Workbench 20.0.3.0 (QIAGEN). The genomic sequences of HCoV-19 obtained in our study have been submitted and stored in China National Microbiology Data Center (NMDC;

http://nmdc.cn/coronavirus; Accession id: NMDC60013485-NMDC60013500)

Sequence retrieval and phylogenetic analysis

We determined the lineage of viral genomes sequenced in this study using PANGOLIN (https://github.com/hCoV-2019/pangolin). We also collected all HCoV-19 genomes from mainland China, the representative sequences of each lineage, sequences of lineage B1.1 in NMDC and GISAID databases (Rambaut et al., 2020). Genomic sequences that are considered to contain many sequencing errors were discarded from our analysis (https://virological.org/t/temporal-signal-and-the-evolutionary-rate-of-2019-n-cov-using-47-genomes-collected-by-feb-01-2020/379). After sequence alignment was performed by MAFFT v7.310 (Katoh et al., 2002), we trimmed the uncertain regions in 3' and 5' terminals (266 and 29674 according to the 1-indexed coordinate of MN908947.3), resulting in total genomic length of 29409 nt. No evidence of recombination was found in our dataset of HCoV-19 genomes in RDP4 (Martin et al., 2015). To understand the phylogenetic and evolutionary relationship among newly isolated HCoV-19 in Beijing in June, other HCoV-19 isolated in mainland China, and virus of lineage B1.1, we reconstructed phylogeny of using GTR substitution model with 1000 ultrafast bootstrap replicates IQ-TREE 2.0.3 (Minh et al., 2020; Hoang et al., 2018).

Evolutionary dynamics analysis

To uncover the potential time of introduction of very recently re-emerging HCoV-19 virus in Beijing in June, 2020, we inferred the time of most recent common ancestor (TMRCA) of the genomes isolated in Beijing. First, we collected all HCoV-19 viruses belonging lineage B1.1 given the lineage information of sequences at https://github.com/cov-lineages/lineages. We discarded redundant sequences and kept genomic sequences with complete collection dates. Sequence alignment and trimming were also performed using the method above. To reduce the computational burden and get more even distribution of genomes, we randomly collected 10 isolates per week from lineage B1.1 (including 8822 sequences). We generated 5 datasets of random selected sequences of lineage B1.1 to avoid the potential biases caused by subsampling process, and each dataset includes 173 genomes with both virus of lineage B1.1 and genomes isolated in Beijing in June, 2020. No evidence of recombination was detected in these datasets by using RDP4. IQ-TREE 2.0.3 was used to reconstruct the phylogeny under GTR substitution model with 1000 ultrafast bootstrap replicates (Hoang et al., 2018). The relationship between root-to-tip divergence (from the phylogeny above) and sampling dates for genomic data was investigated using TempEst v1.5.3 (Rambaut et al., 2016). We used the Bayesian Markov Chain Monte Carlo (MCMC) approach implemented in BEAST v1.10.4 to infer the evolutionary dynamics of HCoV-19 (Suchard et al., 2018). The GTR model, gamma distribution with 4 categories, strict molecular clock model and constant tree priors were used to model evolution of sequences. We performed the analysis over five datasets for each 100 million MCMC steps, sampling parameters and trees every

10,000 steps. Tracer 1.7.1 was then used to check the convergence of MCMC chain (effective sample size >200) and to compute the marginal posterior distributions of parameters, after discarding of 10% of the MCMC chain as burn-in (Rambaut et al., 2018). TreeAnnotator was used to summarize a maximum clade credibility (MCC) tree (Figure 3) from the posterior distribution of trees (after discarding of 10% of the MCMC chain as burn-in).

References

Hoang D.T., Chernomor O., Haeseler A.V., Minh B.Q., Vinh L.S. (2018). UFBoot2: improving the ultrafast bootstrap approximation. Mol. Biol. Evol. 35, 518-522.

Katoh K., Misawa K., Kuma K., Miyata T (2002). MAFFT: a novel method for rapid multiple sequence alignment based on fast Fourier transform. Nucleic Acids Res. 30: 3059-3066.

Martin D.P., Murrell B., Golden M., Khoosal A., Muhire B. (2015). RDP4: detection and analysis of recombination patterns in virus genomes. Virus Evol. 1, vev003.

Minh B.Q., Schmidt H.A., Chernomor O., Schrempf D., Woodhams M.D., Haeseler A.V., Lanfear R. (2020). IQ-TREE 2: new models and efficient methods for phylogenetic inference in the genomic era. Mol Biol Evol. 37, 1530-1534.

Rambaut A., Drummond A.J., Xie D., Baele G., Suchard M.A. (2018). Posterior summarization in Bayesian phylogenetics using Tracer 1.7. Syst. Biol. 67, 901-904.

Rambaut A., Holmes E.C, Hill V, O'Toole Á, McCrone J.T., Ruis C., Plessis L.D., Pybus O.G. (2020). A dynamic nomenclature proposal for SARS-CoV-2 to assist genomic epidemiology. bioRxiv. DOI: https://doi.org/10.1101/2020.04.17.046086.

Rambaut A., Lam T.T., Max Carvalho L., Pybus O.G. (2016). Exploring the temporal structure of heterochronous sequences using TempEst (formerly Path-O-Gen). Virus

Evol. 2, vew007.

Suchard M.A., Lemey P., Baele G., Ayres D.L., Drummond A.J., Rambaut A. (2018). Bayesian phylogenetic and phylodynamic data integration using BEAST 1.10. Virus Evol. 4, vey016.

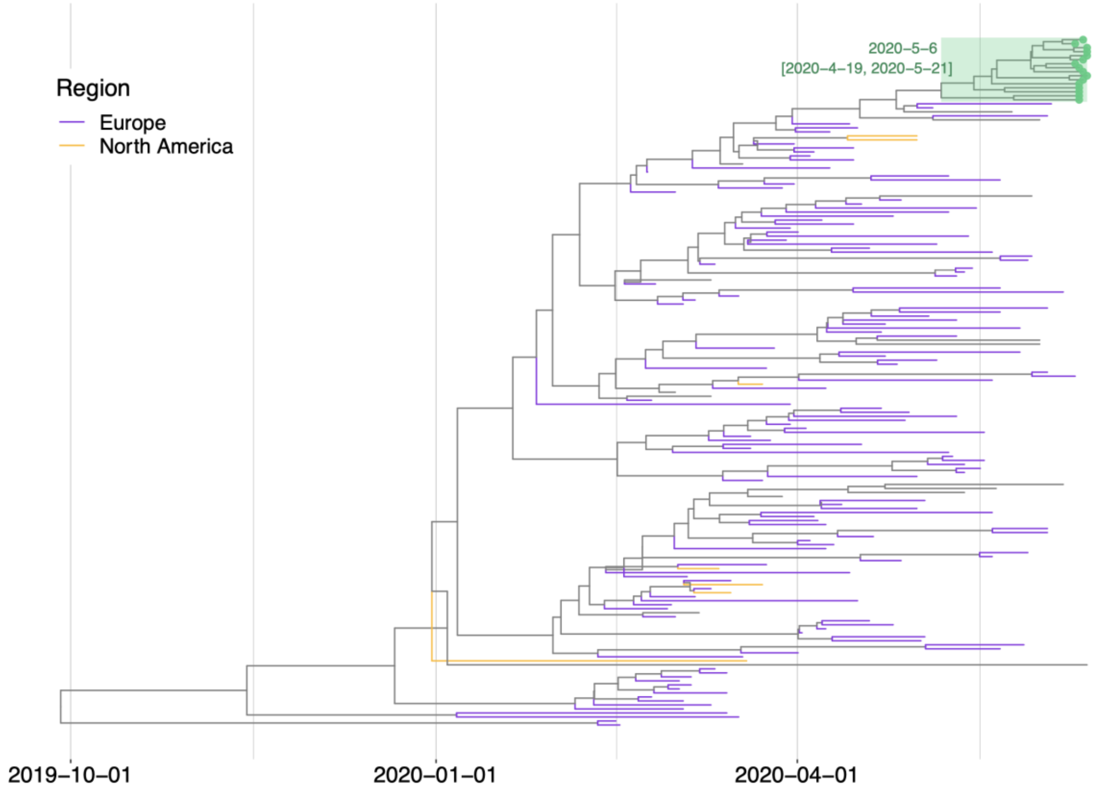

Figure S1. Time-scaled phylogeny of HCoV-19 isolated in the re-outbreak of Beijing in June 2020, mainland China, and randomly selected genomes from lineage B1.1. The isolates collected in Beijing were colored in green. Branches connecting tips are colored according to their isolation locations.

Table 1. Estimates of evolutionary rates and the time of the most recent common ancestor (TMRCA) of the HCoV-19 viruses re-isolated in Beijing in June, 2020.

**Dataset**	**Model**			**Evolutionary rate (substitutes per site)**	**TMRCA**
	**Site model**	**Clock model**	**Tree prior**		
Subsample1	GTR	Strict	Constant	7.07E-04 [5.40E-04, 8.82E-04]	2020-5-6 [2020-4-19, 2020-5-21]
Subsample2	GTR	Strict	Constant	8.88E-04 [6.96E-04, 1.09E-03]	2020-5-12 [2020-4-27, 2020-5-24]
Subsample3	GTR	Strict	Constant	9.55E-04 [7.41E-04, 1.19E-03]	2020-5-14 [2020-4-30, 2020-5-26]
Subsample4	GTR	Strict	Constant	8.83E-04 [6.90E-04, 1.08E-03]	2020-5-10 [2020-4-25, 2020-5-24]
Subsample5	GTR	Strict	Constant	9.11E-04 [7.36E-04, 1.10E-03]	2020-5-11 [2020-4-26, 2020-5-24]
